# Correction: Active sensing in the categorization of visual patterns

**DOI:** 10.7554/eLife.25660

**Published:** 2017-02-06

**Authors:** Scott Cheng-Hsin Yang, Máté Lengyel, Daniel M Wolpert

Yang SC-H, Lengyel M, Wolpert DM. 2016. Active sensing in the categorization of visual patterns. *eLife* **5**:e12215. doi: 10.7554/eLife.12215.

Published 10, February 2016

We would like to thank Greg Sotiropoulos for pointing out to us that the posterior weightings in Equation 9 were mistakenly written as prior weightings.

Equation 9 in the published article read:

(9) $$P\left(z^{*}|x^{*},c=\text{S},D\right)=\frac{1}{2}P\left(z^{*}|x^{*},\theta_{\text{SV}},D\right)+\frac{1}{2}P\left(z^{*}|x^{*},\theta_{\text{SH}},D\right)$$

It should have read:

(9) $$P\left(z^{*}|x^{*},c=\text{S},D\right)=\frac{P\left(D|\theta_{\text{SV}}\right)}{P\left(D|\theta_{\text{SV}}\right)+P\left(D|\theta_{\text{SH}}\right)}P\left(z^{*}|x^{*},\theta_{\text{SV}},D\right)+\frac{P\left(D|\theta_{\text{SH}}\right)}{P\left(D|\theta_{\text{SV}}\right)+P\left(D|\theta_{\text{SH}}\right)}P\left(z^{*}|x^{*},\theta_{\text{SH}},D\right)$$

We apologize for any confusion that may have occurred.

The article has been corrected accordingly.

